# Retraction: Evaluation of the Efficacy of the HaeNaem Zero Bone Loss Kit in Indirect Sinus Lift Using Osseodensification

**DOI:** 10.7759/cureus.r152

**Published:** 2024-11-26

**Authors:** Rachel Changrani, Amod P Patankar, Swapna A Patankar, Pranjali Kulkarni, Amisha Sharma

**Affiliations:** 1 Oral and Maxillofacial Surgery, Bharati Vidyapeeth (Deemed to be University) Dental College and Hospital, Pune, IND; 2 Oral Pathology and Microbiology, Bharati Vidyapeeth (Deemed to be University) Dental College and Hospital, Pune, IND

This article has been retracted by the Editors-in-Chief and its contents removed. This decision is based on the inclusion of the following statement which references a prior study that was not a comparison of the two types of burs: "Moreover, when compared to Densah burs, the OD and indirect sinus lift performed with HaeNaem drills offer a more favorable risk-to-benefit ratio [12]." Importantly, there is no comparison of the alveolar ridge height using the Densah bur and the HaeNaem bur under the same conditions. In addition, it has been discovered that the subject burs were not registered with the CDSCO until November 4, 2024, which is after the study took place. As a result of the above, this article has been retracted and its contents removed.

